# Factors associated with childhood ocular morbidity and blindness in three ecological regions of Nepal: Nepal pediatric ocular disease study

**DOI:** 10.1186/1471-2415-14-125

**Published:** 2014-10-23

**Authors:** Srijana Adhikari, Mohan Krishna Shrestha, Kamala Adhikari, Nhukesh Maharjan, Ujjowala Devi Shrestha

**Affiliations:** Tilganga Institute of Ophthalmology, Kathmandu, Nepal; Freelance, Epidemiologist, Kathmandu, Nepal

**Keywords:** Nepal, Blindness, Pediatric, Ocular

## Abstract

**Background:**

Nepal Pediatric Ocular Diseases Study is a three year longitudinal population based study. Here we present the baseline survey report which aims to investigate various risk factors associated with childhood ocular morbidity and blindness in three ecological regions of Nepal.

**Method:**

This baseline survey is a population based cross sectional study. The investigation was conducted in a district from each of the following regions: Terai, Hill and Mountain. The Village Development Committees (VDCs) from each district were selected by random sampling. Three Community health workers were given training on vision screening and identification of abnormal ocular signs in children. They conducted a house to house survey in their respected districts examining the children and gathering a standardized set of data variables. Children with abnormal vision or ocular signs were then further examined by pediatric ophthalmologists.

**Results:**

A total of 10950 children aged 0–10 years (5403 from Terai, 3204 from the hills, 2343 from the mountains) were enrolled in the study. However 681 (6.2%) were non responders. The male to female ratio was 1.03. The overall prevalence of ocular morbidity was 3.7% (95% CI of 3.4%-4%) and blindness was 0.07% (95% CI of 0.02%-0.12%). Ocular morbidity was more prevalent in the mountain region whereas blindness was more prevalent in the Terai region.

Children from the Terai region were more likely to suffer from congenital ocular anomalies compared to the other regions. Children whose mother smoked, drank alcohol, or was illiterate were significantly afflicted with ocular diseases (p < 0.05). In addition,a higher prevalence of ocular disease was related to children with past medical history of systemic illnesses, abnormal postnatal period or missing childhood vaccinations. Blindness was more prevalent in children who suffered from a systemic illness. Females and under-nourished children were more likely to have ocular morbidity and blindness.

**Conclusion:**

It was found that childhood blindness was more prevalent in the Terai region, the undernourished, females and in those with co-morbid systemic illnesses. This study strongly suggests that prevention of childhood blindness requires additional resources to address these disparity.

## Background

Vision disorders are among the most common disabilities to affect children. It has been estimated that there are 1.4 million blind children worldwide, two third of whom live in the developing 6 countries like Nepal [1]. According to World Health Organization (WHO) childhood blindness is a priority, and significant component of its vision 2020 program [2]. Population based data on the prevalence of childhood ocular morbidity and blindness, which are needed to set priorities and to plan strategies are limited worldwide including in Nepal [3, 4]. These types of studies are important in determining the magnitude of the problem and also to explore associated risk factors, which may be social, environmental and/or biological. A population based study can help establish targeted educational and screening programs in order to reduce known modifiable risk factors. Data on childhood blindness derived from the nationwide survey in the blind schools of Nepal has been previously reported [5]. In Nepal, it has been estimated that there are 30240 blind children and another 120000 children suffering from low vision [6]. There are also other reports addressing ocular morbidity in school children [7–9].

However, a population based survey of childhood ocular morbidity and blindness of all age groups have not yet been assessed in Nepal. Nepal Pediatric Ocular Diseases Study (NPODS) aims to fulfill this void. It is a population based three year longitudinal study which has been designed to evaluate the disease prevalence and incidence in children in a defined population from three geographically diverse districts of Nepal. The baseline survey has been completed and the goal is to report the demographic and biological risk factors associated with childhood ocular morbidity and blindness with emphasis on potentially preventable or modifiable factors.

## Methods

This baseline survey is a population based cross sectional study. The study was approved by the Institutional Review Board of Tilganga Institute of Ophthalmology. It adheres to the tenets of the Declaration of Helsinki.

### The study area

Nepal is divided into three geographically diverse regions: Terai in the south is a flat plane, in the central zone there are hills and in the north, the terrain is mountainous. Each region is subdivided into districts and within the districts are Village Development Committees (VDCs). One district from each region was selected: Sarlahi from the Terai region, Makawanpur from hilly region and Sindhupalchowk from the mountainous region. The number of VDCs selected for each region was in proportion to the sample size: four from the Terai, two from the hills and two from the mountainous region.

### The study population

All the children from 0 to 10 years of age residing in the selected geographical areas were included in the study.

### Sample size and sampling method

For sample size calculation, the formula of estimating a population proportion with specified relative precision was used in this study. Taking the ocular morbidity prevalence of 5%, 10% of relative precision and considering the design effect of 1.5, a total sample size of 10950 was needed for this study. The total sample size was taken from 3 selected districts proportionately according to their projected population in 2011. Based on those 2343 children from the Sindhupalchowk, 3204 from the Makawanpur and 5403 from the Sarlahi were recruited. Three districts from each ecological region were selected by the purposive sampling and the VDCs were selected by the simple random sampling method.

### The field examination

One CHW was recruited from each of the three districts. They were given training on testing age specific visual acuity in children and on detecting abnormal ocular signs using a torch light. A special data collection form was designed and pretested in the similar community in the Kathmandu valley. Informed consent was taken from all the parents of children included in the study.

The CHWs conducted a house to house survey where they took a detailed history and examined each child. The history was taken from the parents and this included a maternal history (antenatal, birth and post natal, literacy, smoking and alcohol), their child’s history (systemic illnesses and any previous ocular surgery or treatment) as well as known family history.

This field examination included the following:

1. Visual acuity was assessed and the method of measuring it was dependent on the age of the child. In children less than 4 years old visual acuity was measured using the fixation preference method with a torch light. Children 4–6 years of age were examined using the HOTV matching test, at three meters distance. In children aged 6 years and older, vision was assessed using the Snellen acuity chart.
2. Height and weight of the child.
3. Torch light examination to assess for five abnormal signs: red eye, head tilt, white pupillary reflex, deviation of eye and watering
4. Motility examination
5. Cover test.

### Examination of abnormal children

Children were subsequently further examined by two pediatric ophthalmologists from the Tilganga Institute of Ophthalmology, if the visual acuity of the child was less than 6/9 in one or both eyes or if they presented with any abnormal ocular signs. The children were brought to an eye clinic close to their homes for this more detailed assessment.

Blindness in this study was defined as a presenting visual acuity of less than 6/60 in the better eye and visual impairment was defined as a presenting visual acuity of less than 6/18 in the better eye. Risk factors were categorized into 3 groups: demographic, maternal and the biological.

### Statistical analysis

Data entry was recorded in Epi data version 3.1. Data analysis was done in the Statistical Package for Social Sciences (SPSS) 16.5 to determine if there was an association between ocular morbidity and blindness with various suspected risk factors. A p value <0.05 was considered significant.

## Results

A total of 10,950 children were enrolled from all three districts. Out of these patients 681 (6.2%) were non responders. Of the remaining participants 50.7% (5208) were from Sarlahi, 30.5% (3136) were from Makawanpur and 18.8% (1926) were from Sindhupalchok. The mean age of the study population was 5.7 (SD 3.1). Thirty seven point eight percent (37.8%) were aged between 0–5 years old and the remainder were aged between 6 – 10 years old. The male to female ratio was 1.03:1. Among the age group of 0–5 years, 39.1% of children were in terai, 40.3% in hills and 30.4% were in mountain region. In the age group 6–10 years, there were 60.9% in terai, 59.7 in hills and 69.6% in the mountain region. The proportion of children in each group varied among the three regions.

### The prevalence of ocular morbidity and blindness

Ocular morbidity was present in 374 children in total and this leads to a prevalence of 3.7% (95% CI 3.3%-4.0%). Only 8 children were afflicted with more than one type of ocular morbidity. The most common ocular problem was conjunctivitis (acute or chronic) and this affected 43% of participants (Table 1).

**Table 1:** **Prevalence of different ocular diseases in three ecological regions**

Pattern of diseases	Total		Terai		Hill		Mountain	
	N	%	N	%	N	%	N	%
Conjunctivitis (Acute and Chronic)	162	43.3	7	6.0	18	23.1	137	76.1
Corneal opacity	81	21.7	53	45.7	16	20.5	12	6.7
Strabismus	50	13.4	12	10.3	16	16.7	22	12.2
Amblyopia	35	9.4	13	11.2	13	16.7	9	5.0
Congenital ptosis	13	3.5	9	7.8	2	2.6	2	1.1
Refractive error	7	1.9	3	2.6	2	2.6	2	1.1
Congenital cataract/aphakia	6	1.6	3	2.6	2	2.6	1	0.6
Choroidal coloboma	4	1.1	4	3.5	0	0.0	0	0.0
Congenital lid and adnexal diseases	5	1.3	0	0.0	3	3.9	2	1.1
Optic atrophy	2	0.5	1	0.9	0	0.0	1	0.6
Retinal diseases	3	0.8	3	2.6	0	0.0	0	0.0
Nystagmus	4	1.1	1	0.9	1	1.3	2	1.1
Globe abnormalities	6	1.6	4	3.5	2	2.6	0	0.0
Others	4	1.1	1	0.9	2	2.6	1	0.6

The prevalence of blindness (defined as a presenting visual acuity (PVA) of less than 6/60) was found to be 0.07% (95% CI 0.02%-0.12%) and visual impairment (PVA less than 6/18, but greater than 6/60 in the better eye) was 0.1% (95% CI of 0.04%-0.15%) (Table 2).

**Table 2:** **Blindness and visual impairment in three ecological regions**

Region	Blindness		Visual impairment	
	Crude (95% CI)	Age adjusted (95% CI)	Crude (95% CI)	Age adjusted (95% CI)
Terai	0.07% (0.02-0.13)	0.08% (0.02-0.13)	0.09% (0.04-0.15)	0.08% (0.02-0.13)
Hill	0.06% (0.01-0.11)	0.07% (0.02- 0.11)	0.16% (0.06-0.24)	0.17% (0.07-0.24)
Mountain	0.05% (0.02-0.11)	0.06% (0.01-0.11)	0	0

In Table 3, it is noted that there is a significant difference (p value <0.05) in the prevalence of ocular disease between the 3 different regions. In descending order of ocular disease prevalence, the mountainous region contained the highest proportion, followed by Terai and then the hill regions.

**Table 3:** **Factors associated with the ocular morbidity in three ecological regions**

Description		Ocular disease present N (%)	Ocular disease absent N (%)	Chi-square test
				P value
**Demographic factors**				
Region	Terai	116(2.2%)	5092(97.7%)	0.000
	Hill	78(2.5%)	3058(97.5%)	
	Mountain	180(9.3%)	1746(90.7%)	
Age (years)	<5	96(2.5%)	3789(97.5%)	0.000
	> = 5-10	278(4.4%)	6107(95.6%)	
Sex	Female	172(3.4%)	4916(96.6%)	0.16
	Male	202(3.9%)	4980(96.1%)	
Religion	Hindu	140(2.7%)	5097(97.3%)	0.000
	Buddhist	184(6.8%)	2523(93.2%)	
	Christian	15(2.5%)	582(97.5%)	
	0thers	35(2.0%)	1694(98.0)	
Type of family	Nuclear	237(3.6%)	6314(96.4%)	0.37
	Joint	117(3.9%)	2890(96.1%)	
	Extended	20(2.8%)	692(97.2%)	
**Maternal factors**				
History of marriage (Consanguinity)	Yes	175(7.0%)	2332(93.0%)	0.000
	No	199(2.6%)	7555(97.4%)	
Mothers education	Literate	117(3.0%)	3733(97.0)	0.006
	Illiterate	257(4.0%)	6163(96%)	
Mother’s smoking habit	Yes	117(3.0%)	3742(97.0%)	0.01
	No	257(4.0%)	6154(96.0%)	
Mother’s alcohol habit	Yes	142(7.1%)	1883(92.9%)	0
	No	2326(2.8%)	8013(97.2%)	
ANC period	Uneventful	339(3.7%)	8774(96.3%)	0.23
	Eventful	35(3.0%)	1122(97.0%)	
**Biological factors**				
Birth history	Full term	373(3.6%)	9885(96.4%)	0.48
	Preterm	1(14.3%)	10(85.7%)	
Place of birth	Home	348(3.7%)	8949(96.3%)	0.9
	HF	26(2.7%)	947(97.3%)	
Type of delivery	Normal	372(3.6%)	9825(96.4%)	0.93
	Forceps	1(4.3%)	22(95.7%)	
	Vacuum	0(0)	3(100%)	
	C/S	1(2.1%)	46(97.9%)	
Postnatal period	Uneventful	370(3.6%)	9881(96.4%)	0.000
	Eventful	4(21.1%)	15(78.9%)	
Immunization	Yes	360(3.5%)	9797(96.5%)	0.000
	No	14(12.4%)	99(87.6%)	
Previous ocular surgery/treatment	Yes	10(21.7%)	36(78.3%)	0.000
	No	364(3.6%)	9860(96.4%)	
Systemic illness	Yes	5(23.8%)	16(76.2%)	0.000
	No	369(3.6%)	9879(96.4%)	

Of interest ocular disease was significantly (p value <0.05) more associated with the following variables: maternal education status, history of consanguinity, smoking and alcohol habit of the mother, children aged 6–10 years old and surprisingly with the Buddhist religion. Other variables investigated were associated with an ocular disease, but the relationship was not significant. Biological risk factors (time of birth, place of birth, type of delivery, postnatal period, immunization, previous ocular surgery, systemic illness) were also studied and many of these were found to be significantly associated with ocular diseases.

The multiple logistic regression analysis was done to determine the association of ocular morbidity with various risk factors. Compared to the Terai region, the likelihood of having an ocular disease in the mountainous region was significantly more than 2 times higher (OR: 2.19, 95% CI: 1.48-3.26), whereas it was non-significantly less likely (OR: 0.79, 95% CI: 0.51-1.22) in the hilly region (Table 4).

**Table 4:** **Association of ocular disease and risk factors (Multiple Logistic regressions for risk factors)**

Risk factors	OR (95% CI)	P value
Region		
Terai	1	-
Hill	0.79(0.51-1.22)	0.295
Mountain	2.19(1.48-3.26)	0.001
Age group (years)		
0-5 years	1	-
>5-10	1.52(1.2-1.92)	0.001
Religion		
Hindu	1	-
Buddhist	1.81(1.32-2.49)	0.001
Christian	1.3(0.71-2.37)	0.4
others	0.88(0.59-1.32)	0.544
Mother’s habit of alcohol intake		
No	1	
Yes	1.22(0.94-1.57)	0.13
Mother’s education status	1	
Literate		
Illiterate	1.37(1.09-1.71)	0.97
Mothers history of marriage		0.04
Consanguinity	1	
Non consanguinity	0.26(0..21-0.33)	
Immunization status		0.001
Immunized	1	
Non-immunized	2.95(1.63-5.36)	
Systemic illness		0.001
Present	1	
Absent	0.1(0.04-0.23)	
Previous ocular surgery/treatment		0.001
Yes	1	
No	0.11(0.06-0.22)	
Postnatal period		0.01
Non eventful	1	
Eventful	5.33(1.45-17.02)	

OR: Odds Ratio, CI confidence interval.

Table 5 shows the association of visual impairment and blindness with measured risk factors. Both visual impairment and blindness were common in patients aged between 6–10 years old. More than two thirds of visual impairment cases were seen in male patients whereas blindness was more common in female children. Sixty percent of visual impairment cases were seen in children who practiced Hinduism and 42.9% of blind cases were prevalent in the other religions.

**Table 5:** **Factors associated with visual impairment and blindness**

Characteristics	Visual impairment		Blindness	
	Yes	No	Yes	No
**Demographic factors**				
Region				
Terai	5(50.0%)	5203(50.7%)	4(57.1%)	5204(50.7%)
Hill	5(50.0%)	3131(30.5%)	2(28.6%)	3134(30.5%)
Mountain	0(0%)	1926(18.8%)	1(14.3%)	1925(18.8%)
Age group				
<5 years	1 (10%)	3884(37.9%)	1(14.3%)	3884(37.8%)
> = 5-10 years	9(90.0%)	6376(62.1)	6(85.7%)	6379(62.2%)
Sex				
Female	2(20.0%)	5086(49.6%)	4(57.1%)	5084(49.5%)
Male	8(80.0%)	5174(50.4%)	3(42.9%)	5179(50.5%)
Religion				
Hindu	6(60.0%)	5231(51.0%)	2(28.6%)	5235(51.0%)
Buddhist	1(10%)	2706(26.4%)	1(14.3%)	2706(26.4%)
Christian	1(10.0%)	596(5.8%)	1(14.3%)	596(5.8%)
0thers	2(20.0%)	1727(16.8%)	3(42.9%)	1726(16.8%)
History of marriage (Consanguinity)				
Yes	0	1766(17.2%)	2(28.6%)	1764(17.2%)
No	10(100%)	8494(82.8%)	5(71.4%)	8499(99.9%)
**Maternal factors**				
Mothers education				
Literate	4(40%)	3846(37.5%)	2(28.6%)	3848(37.5%)
Illiterate	6(60%)	6414(62.5%)	5(71.4%)	6415(62.5%)
Mother’s smoking habit				
Yes	6(60.0%)	6405(62.4%)	5(71.4%)	6406(62.4%)
No	4(40.0%)	3855(37.6%)	2(28.6%)	3857(37.6%)
Mother’s alcohol habit				
Yes	8(80%)	8237(80.3%)	5(71.4%)	8240(80.3%)
No	2(20%)	2023(19.7%)	2(28.6%)	2023(19.7%)
ANC period	*			
Uneventful	6(60.0%)	9107(88.8%)	7(100%)	9106(88.7%)
Eventful	4(40.0%)	1153(11.2%)	0(0)	1157(11.3%)
**Biological factors**				
Birth history				
Full term	10(100%)	10248(99.9%)	7(100%)	10251(99.9%)
Preterm	0(0)	12(.1%)	0(0%)	12(.1%)
Place of birth				
Home	9(90%)	9288(90.5%)	7(100%)	9290(90.5%)
HF	1(10%)	972(9.5%)	0(0%)	973(9.5)
Postnatal period				
Uneventful	10(100%)	10241(99.8%)	7(100%)	10244(99.8%)
Eventful	0(0%)	19(0.2%)	0(0%)	19(0.2%)
Immunization				
Yes	10(100%)	10147(98.9%)	7(0.1%)	10150(98.9%)
No	0(0%)	113(1.1%)	0(0%)	113(1.1%)
Systemic illness	***			
Yes	1(4.8%)	20(95.2%)	0(0%)	21(100%)
No	9(0.1%)	10240(99.9%)	7(0.1%)	10242(99.9%)
Previous ocular surgery/treatment	***		***	
Yes	1(2.2%)	45(97.8%)	4(8.7%)	42(91.3%)
No	9(0.1%)	10215(99.9%)	3(0.1%)	10221(99.9%)

Note: ***p < 0.001, *p < 0.05.

Both visual impairment and blindness were more prevalent in the children whose mother was illiterate and whose mother smoked and drank alcohol. All children with visual impairment and blindness were delivered at full term and had no history of pregnancy and postnatal complications. However, some cases of visual impairment were found in the children with systemic illness (4.8%) and with a previous history of ocular treatment (2.2%). Similarly, blindness was found in 8.7% of children with a previous history of treatment for any eye diseases. There was a significant relationship (p < 0.05) between blind children who had a history of ocular disease. In addition, visually impaired children were significantly associated with a previous history of ocular surgery or treatment, systemic illness or complicated antenatal period.

The association between nutritional status and ocular morbidity was measured. The mean BMI of all the children was 16.3 (SD 5.7). In Terai, 15.21 (SD 5.6), 16.7 (SD 3.7) in hills and 15.7 (SD 8.0) in the mountains. Overall the majority of children (85.8%) were underweight whereas only 2.2% were obese. Among the total children with ocular morbidity, the majority 317 (90.1%) were underweight. Not surprisingly, more than 90% (73 of the 81 cases) of children with the corneal opacities were under nourished (Table 6).

**Table 6:** **Nutritional status and ocular morbidity**

BMI	Frequency	Percent	Presence of ocular disease	Corneal opacity
Under weight (<=17.50 kg/m2)	7741	75.4	296(81.3%)	68(85.0%)
Normal weight (>17.50-22.99 kg/m2)	2023	19.7	56(15.5%)	11(13.5%)
Over weight (23.00-27.99 kg/m2)	310	3.0	10(2.7%)	1(1.2%)
Obesity (>28 kg/m2)	141	1.4	2(0.5%)	0

## Discussion

The causes of ocular morbidity and blindness vary across countries as they have different environmental variables, socioeconomic, geographic and ethnic backgrounds. It may also be affected by the child’s own biological factors and their total wellbeing. Nepal et al. has shown that refractive error is the most common ocular morbidity in children [7]. However there is a regional disparity; Pokhrel et al. and Shrestha et al. showed that children from rural areas are less likely to have refractive error than children from urban areas [8, 10]. These studies however are mainly focused on the prevalence and causes of ocular diseases but not the risk factors associated with them. Jyoti et al. did a nationwide survey in blind schools, showing that corneal blindness due to Vitamin A deficiency is a major cause of childhood blindness [5]. However, unlike our study it was not population based and they mainly highlighted the etiology and did not focus on elucidating the risk factors.

Our study has found that ocular morbidity was more prevalent in the mountainous region. Conjunctivitis was the most common disease in this region. This is similar to other studies conducted in the high mountains of Tibet and India [11, 12]. On the other hand, congenital abnormalities were more common in the flat terrain of the Terai region. Most congenital diseases have a genetic etiology and it is known that there are many families in this region who practice consanguineous marriage suggesting a possible reason for this higher regional prevalence. Further genetic studies are needed to support this statement.

Children aged 6–10 years old had a higher prevalence of ocular morbidity compared to the younger age group, suggesting that an older age is a risk factor. Conjunctivitis is the most common ocular morbidity in our study and is commonly present in children of this age group. It was also found that there was a strong correlation between ocular diseases and the Buddhist community. Given that most Buddhists in Nepal are originally from the mountains and this study highlights that ocular morbidity was higher from the mountainous region, this may explain why 2 ocular morbidity was more prevalent in the Buddhist community compared to others. Further large scale study will be needed to prove causalty.

Among the maternal factors we found that maternal education, history of consanguinity and maternal history of alcohol consumption were strongly associated with ocular morbidity. However there was an inverse relationship with the maternal history of smoking. There are few studies which show a strong correlation between childhood ocular morbidity and the maternal history of smoking which contradict the findings in this study [13–15]. Also, the millennium cohort study [15] found that the mother’s education has an inverse relationship with ocular diseases, again contrary to the results of this study. The history of smoking and alcohol during the antenatal was taken by the field workers (CHW). They had to rely on the information shared by the mother. This recall bias therefore is a limitation of our study.

We found a significant relationship between ocular diseases and the post natal complications such as birth asphyxia, delayed milestones and prematurity. Also there was a relationship between ocular morbidity and systemic illness such as seizure disorder. These findings are similar to Millennium Cohort and Alspac Study [14.15].

While analyzing the risk factors associated with blindness in these children, we found that blindness was more prevalent in the Terai region, in the female population in children of older age group, with a history of consanguinity and with an illiterate mother. However these factors were not statistically significant. There was a strong association between blindness and a previous history of ocular diseases or treatment. Rahi et al. stated that prenatal factors were found in about 60% of blind children, in the blind school of India [16].

In this study, the factors which are not significantly associated may be better evaluated by increasing the sample size. One important finding in our study was the association between nutritional status and ocular 22 diseases. Approximately 75% of children were under-nourished and within this group 81% children had ocular diseases. Also a significant association was found between corneal opacity with a poor nutritional status of the child. Corneal opacities were present in 22% of children who had ocular disorders and 85% of children with corneal opacities were undernourished. The association between the corneal opacities and malnutrition has been well reported in the literature [17, 18]. In Nepal the national Xerophthalmia survey was done in 1985 which showed that the prevalence of corneal opacities was 0.03% [19]. Another study, done by Shakya SR et al. in Eastern Nepal showed that corneal opacities were found in 0.2% of children with nutritional deficiencies [20]. It is known that under-nutrition and its aftermath are still a public health problem in Nepal despite interventional programs.

## Conclusion

Regional disparity is evident in Nepal; ocular morbidity is more common in the mountainous regions, whereas blindness is more prevalent in the Terai region. Many children from the Terai region suffer from congenital abnormalities and this may be related to consanguinity. This needs further genetic analysis. To prevent unnecessary blindness in children, policy makers should prioritize their resources to improve perinatal care and to support nutritional interventions.
